# Factors associated with nurses’ search for training in auriculotherapy: a national cross-sectional study

**DOI:** 10.1590/1980-220X-REEUSP-2024-0196en

**Published:** 2024-11-25

**Authors:** Daniela Dallegrave, Daiana Cristina Wickert, Isabella Goulart Gonçalves, Maria Denise Schimith, Diéssica Roggia Piexak

**Affiliations:** 1 Universidade Federal do Rio Grande do Sul, Escola de Enfermagem e de Saúde Coletiva, Departamento de Assistência e Orientação Profissional, Porto Alegre, RS, Brazil.; 2 Universidade Federal de Santa Maria, Programa de Pós-Graduação em Enfermagem, Santa Maria, RS, Brazil.; 3 Universidade Federal do Rio Grande do Sul, Instituto de Matemática e Estatística, Porto Alegre, RS, Brazil.; 4 Universidade Federal de Rio Grande, Programa de Pós-Graduação em Enfermagem, Rio Grande, RS, Brazil.

**Keywords:** Auriculoterapia, Acupuntura Auricular, Enfermería, Practicantes de la Medicina Tradicional, Salud Pública, Auriculotherapy, Acupuncture, Ear, Nursing, Traditional Medicine Practitioners, Public Health

## Abstract

**Objective:**

To analyze the sociodemographic profile and highlight factors associated with nurses’ search for training in auriculotherapy.

**Method:**

This is a cross-sectional study, with national scope, with 1,154 nurses. The data was collected digitally from June 2021 to January 2022 through a sociodemographic and training characterization questionnaire. Variables were assessed by descriptive and inferential statistics.

**Results:**

301 participants reported having training in auriculotherapy, the majority being women (88.96%), between 40 and 46 years old (26.86%), white (73.67%), born (48.83%) and working (48.16%) in the South region. The majority of nurses who have training in auriculotherapy (63.12%) did not specialize in acupuncture.

**Conclusion:**

Nurses’ professional practice in auriculotherapy is carried out by professionals who have training in professional qualification courses, a fact that is supported by the regulations of public policy and the professional council. Age was a factor associated with the search for training in auriculotherapy, i.e., as nurses age, especially young adults, there is a greater tendency to seek this practice.

## RESUMEN

**Objetivo:**

Analizar el perfil sociodemográfico y resaltar los factores asociados a la búsqueda de formación en auriculoterapia del(a) enfermero(a).

**Método:**

Se trata de un estudio transversal, a nivel nacional, con 1.154 enfermeros(as). Los datos se recolectaron de manera virtual desde junio de 2021 hasta enero de 2022 mediante un cuestionario sociodemográfico y de capacitación. Las variables fueron evaluadas mediante estadística descriptiva e inferencial.

**Resultados:**

301 participantes declararon tener formación en auriculoterapia, siendo la mayoría mujeres (88,96%), entre 40 y 46 años (26,86%), blancas (73,67%), naturales (48,83%) y activas (48,16%) en la región Sur. La mayoría de los(las) enfermeros(as) que tienen formación en auriculoterapia (63,12%) no tienen especialización en acupuntura.

**Conclusión:**

El ejercicio profesional del(a) enfermero(a) en auriculoterapia es realizado por profesionales capacitados en cursos de calificación profesional, hecho que está respaldado por políticas públicas y normas de los consejos profesionales. La edad fue un factor asociado a la búsqueda de formación en auriculoterapia, es decir, a medida que los(las) enfermeros(as) crecen, especialmente los adultos jóvenes, hay mayor tendencia a buscar esta práctica.

## INTRODUCTION

Auriculotherapy has been a relevant practice for the composition of integrative and complementary care in Brazil. It is a minimally or non-invasive method that helps to relieve pain, treat physical problems^(1)^, contribute to signs and symptoms of health treatments^(2)^ and even diagnose clinical conditions, through the stimulation of specific points in the ear. The auricular region is delimited; its constitutional anatomy offers conditions for a safe practice, with minimal or no risk related to idiosyncrasies, idiopathies or iatrogenesis. Evidence does not have a consensus about the origin of auriculotherapy, which has been used for thousands of years in several countries. However, it can be stated that the greatest frequency in the development of the technique and the first registered documents are from China. In this regard, it is considered that auriculotherapy can have a theoretical-philosophical basis in different medical rationalities, as is the case of Traditional Chinese Medicine (TCM), or the use of ear pavilion as a form of treatment in different cultures, as is the case of some ethnic groups of indigenous peoples^(3)^.

Auriculotherapy is a care technique applied to the microsystem of points located on the ear, which act by stimulating somatotopy, which is when a part of the body corresponds to different body regions. Therefore, when the reflex point on the ear is stimulated, it is possible to obtain a symptom relief action in distant parts of the body^(4)^. Although it may be called auricular acupuncture, in general, in Brazil, training in the *lato sensu* modality is not necessary for auriculotherapy professional practice, being constituted of training in free courses, extension courses, continuing education in health or professional qualification.

The effects of auriculotherapy are related to the physical^(1)^, emotional^(5)^ and hormonal context, without contraindications or side effects, widely consolidated in the scientific literature and systematized in a map of evidence on the clinical effectiveness of this practice in the Virtual Health Library on Traditional, Complementary and Integrative Medicines (In Portuguese, BVS/MTCI - *Biblioteca Virtual em Saúde sobre Medicinas Tradicionais, Complementares e Integrativas*).

As of 2016, the Ministry of Health allocated funds for training Primary Healthcare (PHC) professionals for diagnosis and treatment through auriculotherapy, an Integrative and Complementary Health Practice (ICHP) included in the scope of acupuncture^(6)^. This practice encompasses the Brazilian National Policy for Integrative and Complementary Practices (In Portuguese, PNPIC - *Política Nacional de Práticas Integrativas e Complementares*) actions, which aimed to facilitate access to ICHP for Brazilian Health System (In Portuguese, SUS – *Sistema* Ùnico *de Saúde*) users, in which a significant number of auriculotherapy procedures are performed by nurses. In 2022, 1,448,705 auriculotherapy session procedures were performed in the SUS, of which 797,085 were performed by nurse professionals, which accounts for more than 50%^(7)^.

Due to the magnitude of the profession in the use of auriculotherapy in the SUS, little is known about nurses’ search for training in this ICHP. Resolution 739 of February 5, 2024, at the Federal Nursing Council (In Portuguese, COFEN - *Conselho Federal de Enfermagem*), recently recognized auriculotherapy as training through free courses, recommending a minimum workload of 80 hours^(8)^, but without further guidance. A dissertation carried out in 2022 with nurses from Santa Catarina showed that ICHP training courses were considered to have low workload and insufficient content^(9)^. Thus, considering the highlighted gaps, the justifications for this study can be listed from different perspectives:

- The consolidation of public policy depends on the recognition of the practice and practitioners, according to the World Health Organization^(10)^;

- The provision of auriculotherapy in the SUS occurs expressively in PHC and by nurses;

- Training in auriculotherapy is complex, but also relatively simple. Thus, it is worth highlighting that the quality in the systematization of training contributes to understanding the content of courses, which generally follow three epistemological lines, such as TCM, reflexology and biomedicine, making it possible to apply auriculotherapy even without necessarily recognizing the traditional knowledge that supports this knowledge, in line with the aforementioned statement that this training is independent of specialization in acupuncture;

- From the perspective of professional practice of people with a degree in nursing, the leading role of nurses in auriculotherapy is a space to be conquered^(11)^.

The research question of this article is: what is the sociodemographic profile and what are the factors associated with the search for training in auriculotherapy by nurses in Brazil? Due to the need to reflect on the four items listed above, the relevance of the research is justified, which aims to analyze the sociodemographic profile and highlight the factors associated with the search for training in auriculotherapy by nurses.

## METHOD

### Study Design

This is a cross-sectional study, written in accordance with STrengthening the Reporting of OBservational studies in Epidemiology (STROBE) recommendations, derived from the Brazilian National Survey on the Educational and Professional Profile of Integrative Health and Traditional Practices Nurses (In Portuguese, EnfPICS - *Enfermeiros(as) de Saúde Integrativa e Práticas Tradicionais*), with quantitative data collection carried out from June 2021 to January 2022.

The study design is justified by the use of statistics as an instrument to outline the profile of the study population, correlate auriculotherapy with nurses’ social and demographic aspects and study variables that statistically influence the search for training at the ICHP in question.

### Population, Place and Sample

The population consisted of professionals with a nursing degree (inclusion criteria), who did not need to be actively registered with the professional council and could be retired, from all regions of Brazil.

To ensure data reliability, a specific formula was used to determine the minimum number of participants required for the study. Sample size selection followed the following formula^(12)^:


$$n=\frac{X\;.N\;.P\;(1-P)}{d^{2}(N-1)+X^{2}\text{​}.P\;(1-P)}$$

Where:

n = sample size;

X^2^ = chi-square value for 1 degree of freedom at a confidence level of 0.05, which is equal to 3.89 (pre-determined fixed value);

N = population size;

P = the proportion of the population that one wants to estimate (assumed to be 0.50, since this proportion would provide the maximum sample size);

d = the degree of precision expressed as a proportion (0.05).

This rule made it possible to estimate the minimum sample size so that it would be possible to carry out certain statistical procedures, since different procedures present specific needs in terms of the number of participants. Based on the total population, composed of 582,197 Brazilian nurses^(13)^, and applying the formula, the minimum number of participants was 384. In order to provide visibility and representation nationally, researchers from the five regions of Brazil were included. Furthermore, among the strategies for disseminating the research, it was publicized by COFEN, and a request for dissemination was made to all Regional Nursing Councils (In Portuguese, CORENs - *Conselhos Regionais de Enfermagem*) in the country. In this way, all nurses could be invited to participate^(14)^.

### Data Collection

The data was collected through an electronic questionnaire, presented in the *Universidade Federal do Rio Grande do Sul* (UFRGS) LimeSurvey, consisting of 52 questions, 17 of which were to be answered by all nurses, divided into nine questions related to sociodemographic profile and eight questions related to professional profile. The remaining 34 questions were answered specifically by nurses who stated that they had some training in integrative practices, two of which were on general training, 15 on ICHP training (13 quantitative and two qualitative) and 17 on professional practice (12 quantitative and five qualitative). The questionnaire was submitted and adapted from a pilot test, carried out with six participants from different Brazilian regions, for the purposes of language adaptation and cultural adaptation.

The profile of nurses who had training in auriculotherapy was investigated. The variables analyzed were: sex (female/male); age group quartiles; self-reported skin color (white, yellow, indigenous, brown, black); place of birth (by economic macro-region); whether they had a postgraduate degree (no/yes); level of postgraduate education (specialization/residency, academic master’s degree, professional master’s degree, doctoral degree, post-doctoral degree); income (minimum wages); place of work activities (by economic macro-region).

### Data Analysis

The analysis began with data summarization using IBM SPSS Statistics software, with calculation of descriptive statistics for each variable. This stage allowed examining sample sociodemographic characteristics, including data visualization through histograms for a more comprehensive understanding. In addition to this, an exploratory analysis was conducted to better understand data structure and ensure the validity and interpretability of results, considering it as an essential stage before statistical modeling.

Therefore, the logistic regression method was chosen due to its ability to efficiently deal with binary response variables and categorical or continuous predictive variables. This approach allowed modeling the relationship between independent variables and search for training in auriculotherapy, considering the complexity of interactions among these variables. The method was implemented using the Statistical Analysis System (SAS) software, using the PROC FREQ procedure, to examine the distribution of categorical variables, and the PROC LOGISTIC procedure, to adjust the logistic regression model.

In the logistic regression model, independent variables included were age, race and gender, selected through exploratory analysis, whereas the search for training in auriculotherapy was treated as the binary response variable. A variety of statistics were used, such as regression coefficients, confidence intervals and goodness-of-fit measures, including Akaike Information Criterion (AIC) and Bayesian Information Criterion (BIC), to assess the model fit and the association among variables. These analyses were interpreted throughout the study, providing valuable insights for understanding the profile of nurses interested in auriculotherapy.

### Ethical Aspects

The study was approved by the UFRGS Research Ethics Committee, under Certificate of Presentation for Ethical Consideration (In Portuguese, CAAE - *Certificado de Apresentação para Apreciação* Ética) 43306921.6.0000.5347. All ethical precepts were followed, and the Informed Consent Form was made available to participants, with the expression of consent made by checking the box.

## RESULTS

The study included 1,154 professionals with a nursing degree. Among the participants, 301 reported having training in auriculotherapy, of which the majority were female nurses (88.96%), aged between 40 and 46 years (26.86%), white (73.67%), born (48.83%) and working (48.16%) in the South region, with an income of 3 to 4 minimum wages (40.67%). Table 1 shows the data.

**Table 1:** Sociodemographic profile of nurses with a degree in auriculotherapy as an Integrative and Complementary Health Practice – Brazil, 2022.

Variables	Nurses with a degree in auriculotherapy n (%)
**Gender** Female Male	266 (88.96) 33 (11.04)
**Age group (years)** 21 to 32 years 33 to 39 years 40 to 46 years Older than 46 years	69 (21.38) 74 (26.15) 76 (26.86) 64 (22.61)
**Color/race/ethnicity** Yellow White Indigenous Brown Black	4 (1.33) 221 (73.67) 1 (0.33) 65 (21.67) 9 (3.00)
**Origin** Southeast South Northeast North Midwest	81 (27.09) 146 (48.83) 37 (12.37) 10 (3.34) 25 (8.36)
**Region of practice of the profession** Southeast South Northeast North Midwest	78 (26.09) 144 (48.16) 36 (12.04) 6 (2.01) 35 (11.71)
**Income** Up to 2 minimum wages Between 3 and 4 minimum wages Between 5 and 6 minimum wages Between 7 and 8 minimum wages More than 8 minimum wages	23 (7.67) 122 (40.67) 78 (26) 47 (15.66) 30 (10.00)

The results highlight a female bias in seeking training in auriculotherapy among nurses, although this trend needs to be investigated further, due to the female predominance in nursing in general. In addition, the uniform distribution by age group suggests a diverse interest in this therapeutic approach, signaling a growing acceptance and appreciation of the technique throughout their professional career. Regarding racial and ethnic representation, the predominance of white professionals reflects racial inequalities in the nursing profession and by extension access to continuing education. The geographic concentration in the Southeast and South regions may be influenced by socioeconomic and geographic factors in the search for training courses as well as due to a greater concentration of researchers involved in the project in this region, which may have influenced the greater participation of regional participants.

Based on the data analyzed (Table 2), most nurses trained in auriculotherapy have postgraduate degrees. Within this group, the majority completed a specialization or residency course (59.80%), followed by an academic master’s degree (13.29%), professional master’s degree (8.31%), doctoral degree (11.30%) and postdoctoral degree (0.33%). These results highlight the importance of investing in continuing education and specialization for nursing professionals interested in improving their skills and competencies in complementary therapies.

**Table 2:** Professional profile of nurses with a degree in auriculotherapy as an Integrative and Complementary Health Practice – Brazil, 2022.

Variables	Nurses with at degree in auriculotherapy n (%)
**Postgraduate degree** Yes No	280 (93.02) 21 (6.98)
**Postgraduate degree level** No postgraduate degree Specialization/residency Academic master’s degree Professional master’s degree Doctoral degree Postdoctoral degree	21 (6.98) 180 (59.80) 40 (13.29) 25 (8.31) 34 (11.30) 1 (0.33)

Among the 301 nurses with training in auriculotherapy, only 111 (36.88%) reported having also taken a specialization course in acupuncture or TCM.

Introducing logistic regression modeling, the analysis revealed a moderate association between the observed responses and the probabilities predicted by the model. Although agreement statistics, such as Somers’ D, Gamma, and Tau-a, indicated this moderate association, we also identified a significant percentage of disagreement. This suggests that the model is not perfect in its predictions. However, model selection criteria, such as AIC and BIC, demonstrated that the adjusted model provides a good fit to the data, reinforcing the reliability of the model in predicting the search for training in auriculotherapy among nurses.

Based on the results of logistic regression analysis (Table 3), it is inferred that age influences the probability of seeking training in auriculotherapy, as evidenced by the significant p-value (p < 0.05), indicating an explanatory relationship. It is observed that, as nurses age, especially young adults, there is a greater tendency to seek this practice, evidenced by the increase in the odds of seeking it for each unit increase in age.

**Table 3:** Unadjusted and adjusted analysis of factors associated with the search for training in auriculotherapy – Brazil, 2022.

Variable	Standard error	(95%) Confidence Interval	Wald’s chi-square	p-value
**Age**	0.00941	(1.023. 1.061)	18.9534	<0.001
**Gender**	0.3114	(0.534. 1.810)	0.0030	0.9560
**Color**	0.2159	(0.482. 1.124)	2.0144	0.1558

However, no statistically significant evidence was found that gender or race had a significant impact on seeking training in auriculotherapy. Although these factors may influence decisions to seek training, they were not detected in a statistically significant way in this study.

## DISCUSSION

The results of this study highlight the influence of age on nurses’ decision to seek training in auriculotherapy, showing a greater inclination among young adults. These findings are in line with the primary objective of the research, which was to investigate the factors linked to the search for this training among nursing professionals. Considering the current context of auriculotherapy in Brazil, in which the demand for integrative therapeutic methods has grown, especially among younger generations seeking to complement conventional medicine, the positive association between age and the search for this training suggests a greater openness and interest in unconventional practices.

Another point to be considered is that the PNPIC has been published for 18 years, i.e., nurses with more time in training as professionals may not have had information or training in auriculotherapy, even because it was not a topic of interest at the time, which may lead to them currently seeking it late, as our results indicate.

However, it is important to note that, despite the relevance of age in this scenario, the results did not reveal statistically significant associations among gender, race and the search for training in auriculotherapy, inclining towards the investigation of the possible influence of other complex factors that require more detailed investigation. Ultimately, the findings of this study provide valuable insights into the behavioral and contextual determinants of seeking training in auriculotherapy, contributing to developing effective promotion and training strategies in this field.

Regarding the association between training in auriculotherapy and specialization in acupuncture, this study demonstrated that only 111 (36.88%) of nurses declared having training in both. This fact supports COFEN Resolution 585/2018, which establishes and recognizes acupuncture as a specialty and/or professional qualification^(14)^. In this case, it can be stated that nurses who have both training courses are classified as “acupuncture as a specialty”, and training only in auriculotherapy can be considered as “professional qualification in acupuncture”; in this case, related to the microsystem of the ear, with the technique of auricular acupuncture or auriculotherapy.

It is worth highlighting that, among the regulations for nurses’ professional practice using ICHP, acupuncture (which includes auricular acupuncture) is the practice with the highest number of resolutions issued by COFEN, the first of which dates back to 1997. By July 2024, seven resolutions, one decision^(15)^ (recognizing the Brazilian Association of Acupuncturist Nurses and Integrative Practice Nurses (In Portuguese, ABENAH - *Associação Brasileira de Enfermeiros Acupunturistas e Enfermeiros de Práticas Integrativas*)) and three normative opinions (on ozone therapy, essential oils and hypnosis) had been issued^(16,17,18)^. Among the resolutions^(8,19,20,21,22,23,24)^, only one does not mention acupuncture as a nurses’ professional practice. Summing up, of the 11 regulations issued by COFEN, six constitute matters for the organization of nurses’ professional practice in acupuncture, including auricular acupuncture.

In analyzing the matter, COFEN’s Internal Regulations are considered, which define Resolution as a “normative act of exclusive competence of the Plenary of Cofen”, with the effect of disciplining the professional practice in its correct execution^(25)^. The Normative Decisions and Opinions are instruments to instruct procedures for the operation of the COFEN/CORENs system and establish understandings or procedures to be followed by nursing professionals, respectively. In other words, a Normative Opinion aims to determine interpretations of previous regulations; in this case, ICHP as a nurses’ professional activity. Normative Opinions are interpretations and details of Resolutions. The emphasis on these regulatory nomenclatures of the profession represents a certain hierarchy of Resolutions on Normative Decisions and Opinions. For the analysis of this article, it is important to highlight that the six regulations on acupuncture as a nurses’ professional practice are in the form of Resolutions, which reinforces the importance of auricular acupuncture as a nurses’ practice, in line with the findings of the research.

Internationally, taking the American Holistic Nurses Association (AHNA)^(26)^ as a reference, acupuncture as a professional practice for nurses does not have the same prominence. In this case, the AHNA, which offers a training program in holistic nursing, only recognizes acupressure, which uses knowledge of systemic acupuncture and also microsystem acupuncture, such as auricular acupuncture, applying the massage or digital pressure technique without using other instruments, such as needles, seeds, spheres, laser, etc.

It is considered that, in these regulations on acupuncture (including auricular acupuncture), some movements to value the practice inserted in professional practice are evidenced by the change in nomenclature. Examples of this are the texts included “to use Acupuncture autonomously in their professional conduct” and more recently “Acupuncture as a specialty and/or qualification of nursing professionals”.

The first regulation^(20)^, dated 2003, defines acupuncture as a complementary practice to nurses’ work, a wording that was replaced by autonomous practice only in 2008^(22)^. By referring to it as a complementary practice, it reiterates the need for professionals to be linked to some healthcare institution. The recognition of autonomous practice does not require this institutional link, providing the opportunity to practice systemic or microsystems acupuncture in one’s own office.

The term “specialty” is commonly interpreted as a characteristic attributed after completing an academic specialization course, which has its own rules related to qualifications and recognition by educational institutions, as determined by the Ministry of Education. However, specialty can be attributed to professional specialization, which is based on knowledge acquired in practice and professional practice, with validation based on criteria defined according to each situation. In other professions, it is common for specialty to be attributed through a proficiency exam for specialist qualifications, with the application of tests by institutions recognized and recommended by the professional council.

In the case of ICHP as a nurse’s area of specialty, based on COFEN Decision 114 of 2019, the institution capable of recognizing qualifications in Brazil is ABENAH^(15)^. Possible qualifications in this area could be granted based on tests or analyses by an assessment panel, based on criteria for recognizing knowledge. One example of experience that could be analyzed for this type of qualification is in-service training, such as training exchanges^(27)^, in which teaching practices occur through direct supervision of professionals experienced in work practice. In-service teaching, taken as a situation that can be certified as a professional specialty, values practice, practicing the learned activity, learning through work, corroborating the demands of movements that demonstrate against distance learning in nursing.

This recognition of knowledge for the attribution of specialty to a professional is consolidated by the inclusion of the expression “professional qualification of nursing professionals”, in 2018^(24)^, in COFEN regulations, and reiterated, in 2024^(8)^, by Resolution that specifies the type of course that attributes the specialty of some ICHP, such as auriculotherapy.

The same reasoning can be used in the case of nurses who have taken other professional qualification courses related to body microsystems, treated with different acupuncture techniques, such as craniopuncture or head acupuncture, foot reflexology or others.

This study has limitations related to the fact that data collection was carried out during the COVID-19 pandemic, given that nurses were on the front line of care, research, management and planning of health actions. Therefore, it is inferred that this aspect may have influenced the number of respondents, despite being representative for the minimum sample calculated.

The association between factors that may contribute to a nurse seeking training in auriculotherapy is complex, and research biases may occur due to interpretation of quantitative analyses. In these situations, the mixed methods approach could deepen related qualitative factors. The macroproject included a qualitative approach, which was not explored in this article because it did not specifically address the proposed study question.

## CONCLUSION

The sociodemographic profile of nurses trained in auriculotherapy is female, white, born and working in the South region, with an income between 3 and 4 minimum wages, and the majority have postgraduate degrees at the specialization or residency level, but not in acupuncture.

The results highlight age as a significant factor in the decision to pursue training in auriculotherapy. It is important to recognize the limitations of the statistical model used and the complexity of the factors that may influence this choice, including sociocultural and individual aspects. These findings have important implications for promoting and implementing auriculotherapy training programs as well as providing directions for future research on the determinants of this therapeutic practice.

For Brazilian nurses, having training in auriculotherapy is not necessarily associated with having specialization in acupuncture. This fact draws attention to the need for professional council regulations and public policies to regulate nurses’ professional practice in auriculotherapy, which must take this profile into account. Nurses’ professional practice in auriculotherapy is carried out by professionals who have training in professional qualification courses, a fact that is supported by public policy and professional council regulations.

The findings of this study indicate the need to conduct other types of analyses that influence the demand for training in auricular acupuncture by nurses. The research undertaken in the macroproject from which this article was extracted found that nurses undertake ICHP training whenever a free opportunity arises, but the association between the search for training in auriculotherapy and the opportunity for free training was not investigated, as is the case of the course financed by the Ministry of Health, which is responsible for training the majority of healthcare professionals in auriculotherapy in Brazil.

It is recommended that future research adopt longitudinal approaches to deepen the understanding of these determinants, including consideration of other possible influential factors, such as clinical experience and access to educational resources, aiming to provide the development of effective promotion and training strategies in this field.

## References

[1] Moura CC, Chaves ECL, Cardoso ACLR, Nogueira DA, Azevedo C, Chianca TCM (2019). Auricular acupuncture for chronic back pain in adults: a
systematic review and metanalysis. Rev Esc Enferm USP.

[2] Contim CLV, Espírito Santo FH, Moretto IG (2020). Applicability of auriculotherapy in cancer patients: an
integrative literature review. Rev Esc Enferm USP.

[3] Neves ML (2019). Acupuntura auricular e neuromodulação.

[4] Gori L, Firenzuoli F (2007). Ear acupuncture in European traditional medicine. Evid Based Complement Alternat Med.

[5] Corrêa HP, Moura CC, Azevedo C, Bernardes MFVG, Mata LRFP, Chianca TCM (2020). Effects of auriculotherapy on stress, anxiety and depression in
adults and older adults: a systematic review. Rev Esc Enferm USP.

[6] Universidade Federal de Santa Catarina (2024). Formação em Auriculoterapia para Profissionais da Atenção Básica:
informações gerais [Internet].

[7] DataSUS (2024). Produção ambulatorial do SUS – Brasil – por local de atendimento
[Internet].

[8] Conselho Federal de Enfermagem – Cofen (2024). Resolução cofen nº 739 de 05 de fevereiro de 2024. Normatiza a atuação da Enfermagem nas Práticas Integrativas e
Complementares em Saúde [Internet].

[9] Wickert DC (2022). Santa Maria [dissertação].

[10] World Health Organization (2013). WHO traditional medicine strategy: 2014–2023 [Internet].

[11] Aires R, Pinho MCV, Coque A (2023). O enfermeiro na prática da auriculoterapia: um protagonismo a ser
conquistado. BJHR.

[12] Hill MM, Hill A (2002). Investigação por questionário.

[13] Conselho Federal de Enfermagem (2020). Enfermagem em Números [Internet].

[14] Conselho Federal de Enfermagem (2022). Pesquisa vai analisar perfil de enfermeiros de práticas integrativas e
tradicionais [Internet].

[15] Conselho Federal de Enfermagem (2019). Decisão COFEN Nº 114/2019 [Internet].

[16] Conselho Federal de Enfermagem (2021). Parecer de câmara técnica nº 0054/2021/CTLN/DGEP/COFEN
[Internet].

[17] Conselho Federal de Enfermagem (2020). Parecer de câmara técnica nº 34/2020/CTLN/COFEN [Internet].

[18] Conselho Federal de Enfermagem (2020). Parecer normativo nº 001/2020/COFEN [Internet].

[19] Conselho Federal de Enfermagem (1997). Resolução COFEN Nº 197/1997 [Internet].

[20] Conselho Federal de Enfermagem (2015). Resolução COFEN Nº 283/2015 [Internet].

[21] Conselho Federal de Enfermagem (2003). Resolução COFEN Nº 287/2003 [Internet].

[22] Conselho Federal de Enfermagem (2008). Resolução COFEN Nº 326/2008 [Internet].

[23] Conselho Federal de Enfermagem (2018). Resolução COFEN Nº 581/2018.

[24] Conselho Federal de Enfermagem (2018). Resolução COFEN Nº 585/2018 [Internet].

[25] Conselho Federal de Enfermagem (2015). Resolução COFEN Nº 726/2015 [Internet].

[26] American Holistic Nurses Association (2024). https://www.ahna.org/.

[27] Rio Grande do Sul (2013). Secretaria de Saúde do Estado. Resolução CIB 590/2013
[Internet].

