# Supplementary material for: Factors associated with nurses’ search for training in auriculotherapy: a national cross-sectional study
Source: Rev Esc Enferm USP. 2024 Nov 25;58:e20240196. doi: 10.1590/1980-220X-REEUSP-2024-0196en (PMC11623440; doi:10.1590/1980-220X-REEUSP-2024-0196en)
Supplement: Translation

# Translation: Fatores associados à busca das(os) enfermeiras(os) pela formação em auriculoterapia: estudo transversal nacional

**DOI:** 10.1590/1980-220X-REEUSP-2024-0196pt

**Authors:** Daniela Dallegrave, Daiana Cristina Wickert, Isabella Goulart Gonçalves, Maria Denise Schimith, Diéssica Roggia Piexak

**Affiliations:** 1 Universidade Federal do Rio Grande do Sul, Escola de Enfermagem e de Saúde Coletiva, Departamento de Assistência e Orientação Profissional, Porto Alegre, RS, Brasil.; 2 Universidade Federal de Santa Maria, Programa de Pós-Graduação em Enfermagem, Santa Maria, RS, Brasil.; 3 Universidade Federal do Rio Grande do Sul, Instituto de Matemática e Estatística, Porto Alegre, RS, Brasil.; 4 Universidade Federal de Rio Grande, Programa de Pós-Graduação em Enfermagem, Rio Grande, RS, Brasil.

**Keywords:** Auriculoterapia, Acupuntura Auricular, Enfermagem, Profissionais de Medicina Tradicional, Saúde Pública

## Abstract

**Objetivo:**

Analisar o perfil sociodemográfico e evidenciar os fatores associados à busca das(os) enfermeiras(os) pela formação em auriculoterapia.

**Método:**

Trata-se de estudo transversal, de abrangência nacional, com 1.154 enfermeiras(os). Os dados foram coletados virtualmente no período de junho de 2021 a janeiro de 2022 por meio de questionário de caracterização sociodemográfica e de formação. As variáveis foram avaliadas por estatística descritiva e inferencial.

**Resultados:**

Declararam ter formação em auriculoterapia 301 participantes, a maioria sendo mulheres (88,96%), entre 40 e 46 anos (26,86%), brancas (73,67%), naturais (48,83%) e atuantes (48,16%) na região Sul. A maioria das(os) enfermeiras(os) que têm formação em auriculoterapia (63,12%) não realizou especialização em acupuntura.

**Conclusão:**

O exercício profissional de enfermeiras(os) em auriculoterapia é realizado por profissionais que têm formação em cursos de qualificação profissional, fato que é respaldado pelas normativas da política pública e do conselho profissional. A idade foi um fator associado à busca pela formação em auriculoterapia, ou seja, à medida que as(os) enfermeiras(os) envelhecem, especialmente adultas(os) jovens, há uma maior tendência em buscar essa prática.

## INTRODUÇÃO

A auriculoterapia tem sido uma prática relevante para a composição de cuidados integrativos e complementares no Brasil. Trata-se de um método pouco ou nada invasivo que contribui para aliviar dores, tratar problemas físicos^(1)^, contribuir com sinais e sintomas de tratamentos para saúde^(2)^ e até mesmo diagnosticar condições clínicas, por meio da estimulação de pontos específicos na orelha. A região auricular é delimitada; sua anatomia constitucional oferece condições para uma prática segura, com risco mínimo ou nulo relacionado às idiossincrasias, idiopatias ou iatrogenias. As evidências não possuem um consenso acerca da origem da auriculoterapia, que é utilizada há milhares de anos em diversos países. No entanto, pode-se afirmar que a maior frequência no desenvolvimento da técnica e os primeiros documentos registrados são da China. Considera-se, neste sentido, que a auriculoterapia pode ter embasamento teórico-filosófico em diferentes racionalidades médicas, como é o caso mencionado da Medicina Tradicional Chinesa (MTC), ou uso do pavilhão auricular como forma de tratamento em diferentes culturas, como é o caso de algumas etnias de povos originários^(3)^.

A auriculoterapia é uma técnica de cuidado aplicada ao microssistema de pontos localizados na orelha, que têm ação por estímulo de somatotopia, que é quando uma parte do corpo corresponde a diferentes regiões corporais. Sendo assim, quando se estimula o ponto reflexo na orelha, pode-se obter uma ação de alívio de sintomas em partes distantes do corpo^(4)^. Embora possa ser chamada de acupuntura auricular, de modo geral, no Brasil, não é necessária a formação na modalidade *lato sensu* para o exercício profissional da auriculoterapia, constituindo-se em formação em cursos livres, de extensão, de educação permanente em saúde ou de qualificação profissional.

Os efeitos da auriculoterapia estão relacionados ao contexto físico^(1)^, emocional^(5)^ e hormonal, sem contraindicação ou efeito colateral, consolidados amplamente na literatura científica e sistematizados em mapa de evidências sobre a efetividade clínica desta prática na Biblioteca Virtual em Saúde sobre Medicinas Tradicionais, Complementares e Integrativas (BVS/MTCI).

A partir de 2016, o Ministério da Saúde destinou verbas para a formação de profissionais da Atenção Primária à Saúde (APS) para diagnóstico e tratamento por meio da auriculoterapia, uma Prática Integrativa e Complementar em Saúde (PICS) contemplada no escopo da acupuntura^(6)^. Esta prática abrange as ações da Política Nacional de Práticas Integrativas e Complementares (PNPIC), que objetivou facilitar o acesso às PICS aos usuários do Sistema Ùnico de Saúde (SUS), no qual um expressivo número de procedimentos de auriculoterapia é realizado por enfermeiras(os). Em 2022, foram realizados no SUS 1.448.705 procedimentos de sessão de auriculoterapia, sendo que, destes, 797.085 foram realizados por profissionais enfermeiras(os), o que representa mais de 50%^(7)^.

Devido à magnitude da profissão no emprego da auriculoterapia no SUS, pouco se sabe acerca da busca pela formação das(os) enfermeiras(os) nesta PICS. Recente Resolução do Conselho Federal de Enfermagem (COFEN), nº 739, de 05 de fevereiro de 2024, reconheceu a auriculoterapia como capacitação por meio de cursos livres, recomendando carga horária mínima de 80 horas^(8)^, porém sem maiores direcionamentos. Dissertação realizada no ano de 2022 com enfermeiras(os) de Santa Catarina evidenciou que os cursos de formação em PICS foram considerados com baixa carga horária e conteúdo insuficiente^(9)^. Assim, tendo em vista as lacunas destacadas, as justificativas para este estudo podem ser elencadas em diferentes perspectivas:

- A consolidação da política pública depende do reconhecimento da prática e dos praticantes, conforme a Organização Mundial da Saúde^(10)^;

- A oferta de auriculoterapia no SUS ocorre expressivamente na APS e por enfermeiras(os);

- A formação em auriculoterapia é complexa, mas também relativamente simples. Sob este aspecto, cabe destacar que a qualidade na sistematização da formação contribui para a compreensão do conteúdo dos cursos que, geralmente, seguem três linhas epistemológicas, tais como a MTC, a reflexologia e a biomedicina, sendo possível aplicar a auriculoterapia mesmo sem necessariamente reconhecer os saberes tradicionais que sustentam este conhecimento, indo ao encontro da afirmativa supracitada de que essa formação é independente da especialização em acupuntura;

- Na perspectiva do exercício profissional das pessoas com graduação em enfermagem, o protagonismo da(o) enfermeira(o) na auriculoterapia é um espaço a ser conquistado^(11)^.

A questão de pesquisa deste artigo é: qual o perfil sociodemográfico e quais são os fatores associados à busca pela formação em auriculoterapia por enfermeiras(os) no Brasil? Devido à necessidade de refletir sobre os quatro itens dispostos acima, justifica-se a relevância da pesquisa, que tem como objetivo analisar o perfil sociodemográfico e evidenciar os fatores associados à busca das(os) enfermeiras(os) pela formação em auriculoterapia.

## MÉTODO

### Desenho do Estudo

Estudo transversal, redigido de acordo com as recomendações do *STrengthening the Reporting of OBservational studies in Epidemiology* (STROBE), derivado do Inquérito Nacional sobre o Perfil Educacional e Profissional de Enfermeiros(as) de Saúde Integrativa e Práticas Tradicionais (EnfPICS), com coleta de dados quantitativos realizada no período de junho de 2021 a janeiro de 2022.

O desenho do estudo se justifica pela utilização da estatística como instrumento para traçar o perfil da população em estudo, correlacionar a auriculoterapia com os aspectos sociais e demográficos das(os) enfermeiras(os) e estudar variáveis que influenciam estatisticamente a busca por formação na PICS em questão.

### População, Local e Amostra

A população foi constituída por profissionais com diploma de graduação em enfermagem (critério de inclusão), não necessitando ter registro ativo no conselho profissional e podendo estar aposentado, oriundos de todas as regiões brasileiras.

Para garantir a confiabilidade dos dados, foi utilizada fórmula específica para determinar o número mínimo de participantes necessário ao estudo. A seleção do tamanho amostral seguiu a seguinte fórmula^(12)^:


$$n=\frac{X\;.N\;.P\;(1-P)}{d^{2}(N-1)+X^{2}\text{​}.P\;(1-P)}$$

Onde:

n = tamanho da amostra;

X^2^ = valor do qui-quadrado para 1 grau de liberdade ao nível de confiança de 0,05 e que é igual a 3,89 (valor fixo pré-determinado);

N = o tamanho da população;

P = a proporção da população que se deseja estimar (pressupõe-se que seja 0,50, uma vez que esta proporção forneceria o tamanho máximo amostral);

d = o grau de precisão expresso em proporção (0,05).

Esta regra oportunizou estimar o tamanho amostral mínimo, para que fosse possível a realização de determinados procedimentos estatísticos, uma vez que procedimentos diferentes apresentam necessidades específicas de número de participantes. Com base no total da população, composto por 582.197 enfermeiras(os) brasileiras(os)^(13)^, e aplicando-se a fórmula, chegou-se ao número mínimo de 384 participantes. No intuito de visibilidade e representatividade no contexto nacional, contou-se com pesquisadores das cinco regiões do Brasil. Ainda, entre as estratégias de divulgação da pesquisa, a mesma foi publicizada pelo COFEN, e realizou-se solicitação de divulgação a todos os Conselhos Regionais de Enfermagem (CORENs) do país. Desta forma, todas(os) as(os) enfermeiras(os) poderiam ser convidadas(os) a participar^(14)^.

### Coleta de Dados

Os dados foram coletados por meio de questionário eletrônico, apresentado no *LimeSurvey* da Universidade Federal do Rio Grande do Sul (UFRGS), composto por 52 perguntas, sendo 17 para serem respondidas por todas(os) as(os) enfermeiras(os), divididas em nove questões relacionadas ao perfil sociodemográfico e oito questões relacionadas ao perfil profissional. As demais, ou seja, 34 perguntas, foram respondidas especificamente por enfermeiras(os) que afirmaram ter alguma formação em práticas integrativas, sendo duas sobre formação geral, 15 sobre formação em PICS (13 quantitativas e duas qualitativas) e 17 sobre atuação profissional (12 quantitativas e cinco qualitativas). O questionário foi submetido e adaptado a partir de teste piloto, realizado com seis participantes das diferentes regiões brasileiras, para fins de adequação da linguagem e adaptação cultural.

Foi investigado o perfil das(os) enfermeiras(os) que tinham a formação em auriculoterapia. As variáveis analisadas foram: sexo (feminino/masculino); quartis de faixa etária; cor da pele autorreferida (branca, amarela, indígena, parda, preta); naturalidade (por macrorregiões econômicas); se tinha curso de pós-graduação (não/sim); o nível de pós-graduação (especialização/residência, mestrado acadêmico, mestrado profissional, doutorado, pós-doutorado); renda (em salários mínimos); local de atividades laborais (por macrorregião econômica).

### Análise de Dados

A análise foi iniciada com a sumarização dos dados, utilizando o *software* IBM SPSS *Statistics*, com cálculo de estatísticas descritivas para cada variável. Esta etapa permitiu examinar as características sociodemográficas da amostra, incluindo uma visualização de dados por meio de histogramas para uma compreensão mais abrangente. Além disso, foi conduzida uma análise exploratória para entender melhor a estrutura dos dados e garantir a validade e a interpretabilidade dos resultados, considerando-a como uma etapa essencial antes da modelagem estatística.

Dessa forma, optou-se pelo método de regressão logística, devido à sua capacidade de lidar eficientemente com variáveis de resposta binária e preditivas categóricas ou contínuas. Essa abordagem permitiu modelar a relação entre as variáveis independentes e a busca pela formação em auriculoterapia, considerando a complexidade das interações entre essas variáveis. A implementação do método foi realizada por meio do *software Statistical Analysis System* (SAS), utilizando o procedimento PROC FREQ, para examinar a distribuição das variáveis categóricas, e o procedimento PROC LOGISTIC, para ajustar o modelo de regressão logística.

No modelo de regressão logística, as variáveis independentes incluídas foram idade, cor e gênero, selecionadas a partir da análise exploratória, enquanto que a busca pela formação em auriculoterapia foi tratada como a variável de resposta binária. Uma variedade de estatísticas foi utilizada, tais como coeficientes de regressão, Intervalos de Confiança e medidas de ajuste, incluindo Critério de Informação de Akaike (AIC) e Critério de Informação Bayesiano (BIC), para avaliar o ajuste do modelo e a associação entre as variáveis. Essas análises foram interpretadas ao longo do estudo, fornecendo *insights* valiosos para a compreensão do perfil das(os) enfermeiras(os) interessadas(os) em auriculoterapia.

### Aspectos Éticos

O estudo foi aprovado pelo Comitê de Ética em Pesquisa da UFRGS, sob Certificado de Apresentação para Apreciação Ética (CAAE) n^o^ 43306921.6.0000.5347. Todos os preceitos éticos foram seguidos, e o Termo de Consentimento Livre e Esclarecido foi disponibilizado aos participantes, com a manifestação de consentimento realizada por meio de assinalamento de caixa de seleção.

## RESULTADOS

Participaram do estudo 1.154 profissionais com diploma de graduação em enfermagem. Entre os participantes da pesquisa, 301 declararam ter formação em auriculoterapia, dos quais predominaram enfermeiras mulheres (88,96%), entre 40 e 46 anos (26,86%), brancas (73,67%), naturais (48,83%) e atuantes (48,16%) na região Sul, com renda de 3 a 4 salários mínimos (40,67%). Os dados podem ser observados na Tabela 1.

**Tabela 1:** Perfil sociodemográfico das(os) enfermeiras(os) com formação em auriculoterapia como Prática Integrativa e Complementar em Saúde – Brasil, 2022.

Variáveis	Enfermeiras(os) com**formação em auriculoterapia n (%)**
**Gênero** Feminino Masculino	266 (88,96) 33 (11,04)
**Grupo etário (anos)** 21 a 32 anos 33 a 39 anos 40 a 46 anos Mais que 46 anos	69 (21,38) 74 (26,15) 76 (26,86) 64 (22,61)
**Cor/raça/etnia** Amarela(o) Branca(o) Indígena Parda(o) Preta(o)	4 (1,33) 221 (73,67) 1 (0,33) 65 (21,67) 9 (3,00)
**Naturalidade** Sudeste Sul Nordeste Norte Centro-Oeste	81 (27,09) 146 (48,83) 37 (12,37) 10 (3,34) 25 (8,36)
**Região de exercício da profissão** Sudeste Sul Nordeste Norte Centro-Oeste	78 (26,09) 144 (48,16) 36 (12,04) 6 (2,01) 35 (11,71)
**Renda** Até 2 salários mínimos Entre 3 e 4 salários mínimos Entre 5 e 6 salários mínimos Entre 7 e 8 salários mínimos Mais que 8 salários mínimos	23 (7,67) 122 (40,67) 78 (26) 47 (15,66) 30 (10,00)

Os resultados destacam uma inclinação feminina na busca pela formação em auriculoterapia entre enfermeiras(os), embora seja necessário investigar mais profundamente essa tendência, devido à predominância feminina na enfermagem de forma geral. Além disso, a distribuição uniforme por faixa etária sugere um interesse diversificado nessa abordagem terapêutica, sinalizando uma crescente aceitação e valorização da técnica ao longo da carreira profissional. Quanto à representatividade racial e étnica, a predominância de profissionais brancas(os) reflete as desigualdades raciais na profissão de enfermagem e, por extensão, o acesso à educação continuada. A concentração geográfica nas regiões Sudeste e Sul pode ter influência dos fatores socioeconômicos e geográficos na busca por cursos de formação, assim como devido a uma concentração maior de pesquisadoras envolvidas no projeto nesta região, o que pode ter influenciado a maior participação de participantes regionais.

Com base nos dados analisados (Tabela 2), a maioria das(os) enfermeiras(os) com formação em auriculoterapia tem pós-graduação. Dentro desse grupo, a maioria realizou curso de especialização ou residência (59,80%), seguida por mestrado acadêmico (13,29%), mestrado profissional (8,31%), doutorado (11,30%) e pós-doutorado (0,33%). Esses resultados destacam a importância do investimento em educação permanente e especialização para os profissionais de enfermagem interessados em aprimorar suas habilidades e competências em terapias complementares.

**Tabela 2:** Perfil profissional das(os) enfermeiras(os) com formação em auriculoterapia como Prática Integrativa e Complementar em Saúde – Brasil, 2022.

Variáveis	Enfermeiras(os) com formação em auriculoterapia n (%)
**Pós-graduação** Sim Não	280 (93,02) 21 (6,98)
**Nível de pós-graduação** Sem pós-graduação Especialização/residência Mestrado acadêmico Mestrado profissional Doutorado Pós-doutorado	21 (6,98) 180 (59,80) 40 (13,29) 25 (8,31) 34 (11,30) 1 (0,33)

Entre as(os) 301 enfermeiras(os) com formação em auriculoterapia, apenas 111 (36,88%) declararam também ter realizado curso de especialização em acupuntura ou em MTC.

Introduzindo a modelagem de regressão logística, a análise revelou uma associação moderada entre as respostas observadas e as probabilidades previstas pelo modelo. Embora as estatísticas de concordância, como Somers’ D, Gamma e Tau-a, tenham indicado essa associação moderada, também identificamos um percentual significativo de discordância. Isso sugere que o modelo não é perfeito em suas previsões. No entanto, os critérios de seleção de modelo, como AIC e BIC, demonstraram que o modelo ajustado oferece um bom arranjo aos dados, reforçando a confiabilidade do modelo na previsão da busca pela formação em auriculoterapia entre enfermeiras(os).

Com base nos resultados da análise de regressão logística (Tabela 3), infere-se que a idade influencia a probabilidade de buscar formação em auriculoterapia, como evidenciado pelo valor de p significativo (p<0,05), indicando uma relação explicativa. Observa-se que, à medida que as(os) enfermeiras(os) envelhecem, especialmente adultos jovens, há uma maior tendência em buscar essa prática, evidenciada pelo aumento nas chances de busca para cada aumento unitário na idade.

**Tabela 3:** Análise bruta e ajustada dos fatores associados à busca pela formação em auriculoterapia – Brasil, 2022.

Variável	Erro padrão	Intervalo de Confiança (95%)	Qui-quadrado de Wald	Valor de p
**Idade**	0,00941	(1.023, 1.061)	18,9534	<0,001
**Gênero**	0,3114	(0.534, 1.810)	0,0030	0,9560
**Cor**	0,2159	(0.482, 1.124)	2,0144	0,1558

No entanto, não foram encontradas evidências estatisticamente significativas de que o gênero ou a cor tenham um impacto significativo na busca pela formação em auriculoterapia. Embora esses fatores possam influenciar as decisões de busca pela formação, não foram detectados de forma estatisticamente significativa neste estudo.

## DISCUSSÃO

Os resultados deste estudo destacam a influência da idade na decisão das(os) enfermeiras(os) em buscar formação em auriculoterapia, evidenciando uma maior inclinação entre adultos jovens. Esses achados estão alinhados com o objetivo primordial da pesquisa, que era investigar os fatores ligados à busca por essa formação entre profissionais de enfermagem. Considerando o atual contexto da auriculoterapia no Brasil, em que a procura por métodos terapêuticos integrativos tem crescido, especialmente entre as gerações mais jovens que buscam complementar a medicina convencional, a associação positiva entre idade e busca por essa formação sugere uma maior abertura e interesse por práticas não convencionais.

Outro ponto a ser considerado é que a PNPIC possui 18 anos desde a sua primeira publicação, ou seja, enfermeiras(os) com mais tempo de formação como profissionais talvez não tiveram informações ou formações em auriculoterapia, até por não ser temática de interesse para a época, podendo levar com que atualmente busquem-nas tardiamente, conforme nossos resultados indicam.

No entanto, é importante notar que, apesar da relevância da idade nesse cenário, os resultados não revelaram associações estatisticamente significativas entre gênero, cor e busca pela formação em auriculoterapia, inclinando à investigação da possível influência de outros fatores complexos que exigem uma investigação mais detalhada. Em última análise, os achados deste estudo fornecem *insights* valiosos sobre os determinantes comportamentais e contextuais da busca pela formação em auriculoterapia, contribuindo para o desenvolvimento de estratégias eficazes de promoção e capacitação nesse campo.

Com relação à associação entre formação em auriculoterapia e especialização em acupuntura, este estudo demonstrou que apenas 111 (36,88%) das(os) enfermeiras(os) declararam ter formação em ambos. Este fato corrobora a Resolução COFEN nº 585/2018, que estabelece e reconhece a acupuntura como especialidade e/ou qualificação profissional^(14)^. Neste caso, pode-se afirmar que as(os) enfermeiras(os) que têm ambas as formações estão enquadradas(os) na “acupuntura como especialidade”, e a formação apenas em auriculoterapia pode ser considerada como “qualificação profissional em acupuntura”; neste caso, relacionada ao microssistema do pavilhão auricular, com a técnica de acupuntura auricular ou auriculoterapia.

Cabe destacar que, entre as regulamentações para o exercício profissional de enfermeiras(os) com a utilização das PICS, a acupuntura (que inclui a acupuntura auricular) é a prática com maior número de resoluções emitidas pelo COFEN, sendo que a primeira data de 1997. Até julho de 2024, foram emitidas sete resoluções, uma decisão^(15)^ (reconhecendo a Associação Brasileira de Enfermeiros Acupunturistas e Enfermeiros de Práticas Integrativas (ABENAH)) e três pareceres normativos (sobre ozonioterapia, óleos essenciais e hipnose)^(16,17,18)^. Entre as resoluções^(8,19,20,21,22,23,24)^, apenas uma não menciona a acupuntura como prática profissional da(o) enfermeira(o). Em resumo, das 11 regulamentações emitidas pelo COFEN, seis constituem matéria para a organização do exercício profissional das(os) enfermeiras(os) em acupuntura, incluindo a acupuntura auricular.

Em análise sobre a matéria, considera-se o Regimento Interno do COFEN, que define Resolução como “ato normativo de competência exclusiva do Plenário do Cofen”, com efeitos de disciplinar o exercício profissional em sua correta execução^(25)^. Já a Decisão e o Parecer Normativo são instrumentos para instruir procedimentos para o funcionamento do sistema COFEN/CORENs e fixar entendimentos ou procedimentos a serem seguidos aos profissionais de enfermagem, respectivamente. Ou seja, o Parecer Normativo tem como objetivo determinar interpretações de regulamentações anteriores; neste caso, das PICS como atuação profissional de enfermeiras(os). Os Pareceres Normativos se tratam de interpretações e detalhamentos de Resoluções. O destaque para essas nomenclaturas regulatórias da profissão representa uma certa hierarquia das Resoluções sobre as Decisões e os Pareceres Normativos. Para a análise deste artigo, importa destacar que as seis regulamentações sobre a acupuntura como exercício profissional de enfermeiras(os) são em caráter de Resolução, o que reforça a importância da acupuntura auricular como prática de enfermeiras(os), indo ao encontro dos achados da pesquisa.

Internacionalmente, tomando como referência a *American Holistic Nurses Association* (AHNA)^(26)^, a acupuntura como prática profissional das(os) enfermeiras(os) não dispõe do mesmo destaque. Neste caso, a AHNA, que oferece um programa de formação em enfermagem holística, reconhece apenas a prática de acupressão, que utiliza os conhecimentos da acupuntura sistêmica e também a acupuntura de microssistemas, como é o caso da acupuntura auricular, aplicando a técnica de massagem ou pressão digital sem utilização de outros instrumentos, como agulhas, sementes, esferas, laser, etc.

Considera-se que, nessas regulamentações sobre acupuntura (incluída a auricular), alguns movimentos de valorização da prática inserida na atuação profissional são evidenciados pela mudança de nomenclatura. Exemplos disso são as redações incluídas “usar autonomamente a Acupuntura em suas condutas profissionais” e mais recentemente “a Acupuntura como especialidade e/ou qualificação do profissional de Enfermagem”.

A primeira regulamentação^(20)^, datada de 2003, define a acupuntura como prática complementar ao trabalho da(o) enfermeira(o), redação que passa a ser substituída por prática autônoma somente em 2008^(22)^. Ao referir como prática complementar, reitera a necessidade de a(o) profissional estar vinculada(o) a alguma instituição assistencial. O reconhecimento da prática autônoma prescinde desta vinculação institucional, oportunizando a prática de acupuntura sistêmica ou de microssistemas em consultório próprio.

O termo “especialidade” tem interpretação no senso comum como caraterística atribuída após a realização de um curso de especialização acadêmica, que tem regramentos próprios relacionados à titulação e reconhecimento das instituições formadoras, conforme determinado pelo Ministério da Educação. No entanto, a especialidade pode ser atribuída à especialização profissional, que é baseada nos conhecimentos adquiridos na prática e no exercício profissional, com validação a partir de critérios definidos conforme cada situação. Em outras profissões, é comum que a especialidade possa ser atribuída por meio de exame de suficiência para titulação de especialistas, com a aplicação de provas por instituições reconhecidas e recomendadas pelo conselho profissional.

No caso das PICS como área de especialidade da(o) enfermeira(o), a partir da Decisão COFEN nº 114, de 2019, a instituição apta ao reconhecimento de títulos no Brasil é a ABENAH^(15)^. Possíveis atribuições de titulação nesta área poderiam ser conferidas a partir de aplicação de provas ou análises por banca avaliativa, a partir de critérios para reconhecimento de saberes. Um dos exemplos de experiência possível de ser analisada para este tipo de titulação são as formações em serviço, como é o caso dos intercâmbios formativos^(27)^, em que práticas de ensino ocorrem por meio de supervisão direta de profissionais experientes na prática laboral. O ensino em serviço tomado como situação passível de ser certificada como especialidade profissional valoriza a prática, o praticar a atividade aprendida, a aprendizagem por meio do trabalho, corroborando a reivindicação de movimentos que se manifestam contra a formação à distância na enfermagem.

Esse reconhecimento do conhecimento para atribuição de especialidade a um profissional é consolidado pela inclusão da expressão “habilitação profissional do profissional de enfermagem”, em 2018^(24)^, em regulamentação do COFEN, e reiterada, em 2024^(8)^, por Resolução que especifica o tipo de curso que atribui a especialidade de algumas PICS, como a auriculoterapia.

O mesmo raciocínio pode ser empregado no caso de enfermeiras(os) que realizaram outros cursos de qualificação profissional relacionados a microssistemas do corpo, tratado com diferentes técnicas da acupuntura, como é o caso da craniopuntura ou acupuntura de cabeça, da reflexologia podal ou outros.

Este estudo comporta limitações relacionadas à coleta de dados ter sido realizada durante a pandemia de COVID-19, tendo em vista que as(os) enfermeiras(os) estavam na linha de frente dos cuidados, pesquisa, gestão e planejamento de ações em saúde. Portanto, infere-se que tal aspecto pode ter influenciado o número de respondentes, apesar de representativo para a amostra mínima calculada.

A associação entre fatores que podem contribuir para um(a) enfermeiro(a) buscar pela formação em auriculoterapia é complexa, podendo ocorrer vieses de pesquisa ocasionados pela interpretação de análises quantitativas. Nestas situações, a abordagem de métodos mistos poderia aprofundar fatores qualitativos relacionados. O macroprojeto incluiu abordagem qualitativa, a qual não foi explorada neste artigo porque não abordou especificamente a questão de estudo proposta.

## CONCLUSÃO

O perfil sociodemográfico das(os) enfermeiras(os) com formação em auriculoterapia é de mulheres, brancas, naturais e atuantes na região Sul, com renda entre 3 e 4 salários mínimos, sendo que a maioria possui pós-graduação em nível de especialização ou residência, porém não em acupuntura.

Os resultados destacam a idade como um fator significativo na decisão de buscar formação em auriculoterapia. É importante reconhecer as limitações do modelo estatístico utilizado e a complexidade dos fatores que podem influenciar essa escolha, incluindo aspectos socioculturais e individuais. Essas descobertas têm implicações importantes para a promoção e implementação de programas de formação em auriculoterapia, além de fornecer direcionamentos para futuras pesquisas sobre os determinantes dessa prática terapêutica.

Para enfermeiras(os) brasileiras(os), ter formação em auriculoterapia não necessariamente está associado a ter especialização em acupuntura. Este fato chama atenção para a necessidade de normativas do conselho profissional e das políticas públicas para regularização da atuação profissional de enfermeiras(os) em auriculoterapia, que devem levar em consideração esse perfil. O exercício profissional de enfermeiras(os) em auriculoterapia é realizado por profissionais que têm formação em cursos de qualificação profissional, fato que é respaldado pelas normativas da política pública e do conselho profissional.

Os achados deste estudo indicam a necessidade de realizar outros tipos de análises que influenciem a procura pela formação em acupuntura auricular por enfermeiras(os). A investigação empreendida no macroprojeto do qual este artigo foi extraído constatou que as(os) enfermeiras(os) realizam formações em PICS sempre que aparece uma oportunidade gratuita, mas não foi investigada a associação entre busca por formação em auriculoterapia e a oportunidade de formação gratuita, como é o caso do curso financiado pelo Ministério da Saúde, que é responsável pela formação da maioria dos profissionais de saúde em auriculoterapia no Brasil.

Recomenda-se que pesquisas futuras adotem abordagens longitudinais para aprofundar a compreensão desses determinantes, incluindo a consideração de outros possíveis fatores influentes, como experiência clínica e acesso a recursos educacionais, visando fornecer o desenvolvimento de estratégias eficazes de promoção e capacitação nesse campo.
